# Clinical performance of lung ultrasound in predicting time-dependent changes in lung aeration in ARDS patients

**DOI:** 10.1007/s10877-022-00902-5

**Published:** 2022-08-08

**Authors:** Costamagna Andrea, Steinberg Irene, Pivetta Emanuele, Arina Pietro, Veglia Simona, Brazzi Luca, Fanelli Vito

**Affiliations:** 1 Department of Anaesthesia and Critical Care, AOU Città Della Salute E Della Scienza Di Torino, University of Turin, Corso Dogliotti 14, 10126 Turin, Italy; 2 Department of Surgical Sciences, University of Turin, Turin, Italy; 3 Division of Emergency Medicine and High Dependency Unit, Department of Medical Sciences, AOU Città Della Salute E Della Scienza Di Torino, Turin, Italy; 4 Division of Medicine, UCL, Bloomsbury Institute for Intensive Care Medicine, Gower street, London, UK; 5 Department of Diagnostic Imaging and Radiotherapy, AOU Città Della Salute E Della Scienza Di Torino, University of Turin, Turin, Italy

**Keywords:** Lung ultrasound, ARDS, Point of care ultrasound, Bedside diagnostic tests

## Abstract

To evaluate whether lung ultrasound is reliable bedside tool to monitor changes of lung aeration at the early and late stages of ARDS. LUS was performed in ARDS patients that underwent at least two consecutive CT scan at ICU admission and at least 1 week after admission. Twelve fields were evaluated and graded from 0 (normal) to 3 (consolidation). Changes of LUS score in twelve fields (ΔLUS_tot_) and in four ventral (ΔLUS_V_), intermediate (ΔLUS_I_) and dorsal (ΔLUS_D_) zones were calculated at each time points. Three categories were described: Improve (ΔLUS < 0), Equal (ΔLUS = 0) or Worse (ΔLUS > 0). LUS scores were correlated with total changes in lung CT aeration (ΔCT_air_) and with normally, poorly and not aerated regions (ΔCT_norm_, ΔCT_poor_ and ΔCT_not_, respectively). Eleven patients were enrolled. ΔLUS_tot_ had significant correlation with ΔCT_air_ (r = − 0.74, p < 0.01). ΔLUS_V_, ΔLUS_I_ and ΔLUS_D_ showed significant correlations with ΔCT_air_ (r = − 0.66, r = − 0.69, r = − 0.63, respectively; p < 0.05). Compared to Equal, Improve and Worse categories had significantly higher (p < 0.01) and lower (p < 0.05) ΔCTair values, respectively. Compared to Equal, Improve and Worse categories had lower (p < 0.01) and higher (p < 0.01) ΔCT_not_ values, respectively. LUS score had a good correlation with lung CT in detecting changes of lung aeration.

**Supplementary Information:**

The online version contains supplementary material available at 10.1007/s10877-022-00902-5.

## Introduction

Chest Computed tomography (CT) is the reference imaging technique for the identification and characterization of lung parenchyma anatomical alterations [1]. In the context of Acute Respiratory Distress Syndrome (ARDS), it has been employed to quantify the loss of tissue aeration [2] due to the non-cardiogenic increase in extravascular lung water [3]. Moreover, CT scan allows to identify ARDS morphology and thus the potential for lung recruitment [2, 4]. In ARDS patients, lung ultrasound (LUS), as compared with CT scan, has shown to be able to assess the loss of lung aeration [5] and to predict ARDS morphology [6]. The role of LUS in following-up on aeration changes over time in this population has not been investigated. Therefore, in the context of a study assessing the accuracy of LUS in identifying ARDS morphology [6], we performed a time course analysis to explore the potential role of LUS in monitoring gain or loss of lung aeration as compared to the gold standard (CT scan). We hypothesized that LUS performed at the bedside would accurately quantify lung aeration changes in ARDS patients over time. The primary endpoint was the correlation between LUS score variations and changes in CT scan percentage of aeration over time. The second end point was to quantify CT scan percentage of aeration changes when LUS improved, worsened or remained the same at early and late stages of ARDS.

## Materials and methods

### Subjects

Patients admitted to the Turin university hospital’s respiratory intensive care unit (ICU) for ARDS, [7] with an expected requirement for mechanical ventilation of at least 24 h and undergoing chest CT for clinical assessment were enrolled. Patients < 18 years of age, those with a confirm diagnosis of pulmonary fibrosis or with an expected survival of less than 24 h were excluded from the study. Time course analysis of LUS accuracy in detecting aeration change was performed in patients that for clinical reason underwent at least two CT scan with the appropriate timing, defined as follows: Early (ICU admission) and Late (at least 1 week after). The study was approved by the local Ethics Committee (0117126) and informed consent was obtained according to Italian regulation.

### Study protocol

CT scan at ICU entry was required for enrollment and subsequent repeating of the exam was performed based on clinical assessment of treating physicians. Whenever CT was performed, LUS examination was recorded, maintaining the same ventilatory settings and sedation level.

Bedside US evaluation were performed by two observers, trained by board-certified consultants with expertise in point of care ultrasound, as to reach the minimum requirements defined to perform with accuracy a standard bedside lung US [8–10].

### Lung ultrasound

Using the curvilinear transducer (5–3 MHz), with a Mylab Seven ultrasound machine (Esaote S.p.A, Genova, Italy), all the patients were examined in supine position [11]. Twelve LUS fields, six in the left and six in the right hemithorax, were explored basing on the following landmarks [11, 12]: right and left 2nd to 3rd intercostal (IC) spaces across the mid-clavicular (MC) line (fields 1 and 7, respectively); right and left 5th to 6th IC spaces across the MC line (fields 2 and 8); right and left 3rd to 4th IC space across the anterior axillary (AA) line (fields 3 and 9); right and left 6th to 7th IC space across the AA line (fields 4 and 10); right and left 4th to 5th IC space across the posterior axillary (PA) line (fields 5 and 11); right and left 7th to 8th IC space across the PA line and above the diaphragmatic dome (fields 6 and 12). Three regions were identified: ventral (V), intermediate (I) and dorsal (D), corresponding to the zones 1-2-7-8, 3-4-9-10 and 5-6-11-12, respectively. The ultrasound beam was directed transversally along the intercostal space, to insonate the majority of the corresponding lung region as seen on an axial plane [12]. Display depth was set as ≥ 12 cm to correctly interpret US artifacts. The examination lasted the time necessary to give a real time evaluation of the 12 fields. A detailed description of lung regions and anatomical landmarks has been previously reported [6].

According to LUS image characteristics each field was graded as: N (normal aeration): lung sliding/lung pulse with A lines or less than two B lines for intercostal space; B1 (moderate loss of lung aeration): multiple spaced B-lines, more or equal than 3 for each space; B2 (severe loss of lung aeration): multiple coalescent B lines with or without subpleural consolidations; C (consolidation): presence of a tissue pattern with or without air bronchograms [13–17], where N = 0, B1 = 1, B2 = 2, C = 3 [15]. LUS score was directly obtained (real time) and reported by the observer for each field in a dedicated case report form.

The sum of the LUS scores obtained in every 12 fields defined the LUS_tot_ variable. The sum of the LUS scores obtained in the four ventral (1-2-7-8), intermediate (3-4-9-10) and dorsal (5-6-11-12) fields defined the variables LUS_V_, LUS_I_ and LUS_D_. In order to evaluate lung aeration over time, the relative changes of aeration score in each field (ΔLUS) was calculated for each patient as follows:

$$\Delta {\text{LUS}} = {\text{LUS}}_{{\left( {{\text{Late}}} \right)}} - {\text{LUS}}_{{\left( {{\text{Early}}} \right)}}$$

where LUS_(Early)_ was the LUS at study entry and LUS_(Late)_ was the LUS obtained at least at one week after. In the same way, the relative changes of LUS_tot_, LUS_V_, LUS_I_ and LUS_D_ scores were calculated for each patient as follows:

$$\Delta {\text{LUS}}_{{{\text{tot}}}} = {\text{LUS}}_{{{\text{tot}}\left( {{\text{Late}}} \right)}} - {\text{LUS}}_{{{\text{tot}}\left( {{\text{Early}}} \right)}}$$

$$\Delta {\text{LUS}}_{{\text{V}}} = {\text{LUS}}_{{{\text{V}}\left( {{\text{Late}}} \right)}} - {\text{LUS}}_{{{\text{V}}\left( {{\text{Early}}} \right)}}$$

$$\Delta {\text{LUS}}_{{\text{I}}} = {\text{LUS}}_{{{\text{I}}\left( {{\text{Late}}} \right)}} - {\text{LUS}}_{{{\text{I}}\left( {{\text{Early}}} \right)}}$$

$$\Delta {\text{LUS}}_{{\text{D}}} = {\text{LUS}}_{{{\text{D}}\left( {{\text{Late}}} \right)}} - {\text{LUS}}_{{{\text{D}}\left( {{\text{Early}}} \right)}}$$

Basing on lung aeration changes identified by ΔLUS, three categories has been described:

1. “Improve” category: lung aeration improved if ΔLUS was < 0
2. “Equal” category: lung aeration did not change ΔLUS was = 0
3. “Worse” category: lung aeration worsened if ΔLUS was > 0

### Lung computed tomography

CT scan was performed at study entry and repeated based on clinical evaluation. Lung aeration at CT scan was assessed blindly with quantitative analysis using a dedicated software (Maluna, University of Mannheim, Germany) [18, 19] in twelve regions of interests (ROI), corresponding to the left and right lung in six CT slices, identified as the areas corresponding to the LUS fields based on pre-defined anatomical landmarks. Sternal manubrium apex/ clavicle: zones 1 (right) and 7 (left); pulmonary trunk, 2 cm below the tracheal carina: zones 2 (right) and 8 (left); lower third of the sternal manubrium, 2 cm beneath the zones 1 and 7, in correspondence with the aortic arch and the scapula: zones 3 (right) and 9 (left); the middle of the sternal body, at the heart base: zones 4 (right) and 10 (left); the lower third of the sternal manubrium, in correspondence with the tracheal carina: zones 5 (right) and 11 (left) and 2 cm above the diaphragm were zones 6 (right) and 12 (left) [6]. The analysis of the selected region of interest (ROIs) with the Maluna software is based on the ray attenuation of each pixel expressed in Hounsfield units (HU) and defined as: hyperinflated (− 900 and − 1000 HU); normally aerated (− 900 and − 500 HU); poorly aerated (− 500 and − 100 HU); and non-aerated (− 100 and 100 HU) [19]. Percentage of aeration (P_air_) was derived from Hounsfield units for each ROI, as follows:

$${\text{P}}_{{{\text{air}}}} = \frac{{{\text{Vol}}_{{{\text{air}}}} }}{{{\text{Vol}}_{{{\text{tot}}}} }} \times 100$$

where Vol_tot_ represents the total volume (in mL) and Vol_air_ the volume occupied by air (in mL) in the considered ROI. Percentage of normally (P_norm_), poorly (P_poor_) and not (P_not_) aerated lung where derived as follows:

$${\text{P}}_{{{\text{norm}}}} = \frac{{{\text{Vol}}_{{{\text{norm}}}} }}{{{\text{Vol}}_{{{\text{tot}}}} }} \times 100$$

$${\text{P}}_{{{\text{poor}}}} = \frac{{{\text{Vol}}_{{{\text{poor}}}} }}{{{\text{Vol}}_{{{\text{tot}}}} }} \times 100$$

$${\text{P}}_{{{\text{not}}}} = \frac{{{\text{Vol}}_{{{\text{not}}}} }}{{{\text{Vol}}_{{{\text{tot}}}} }} \times 100$$

where Vol_norm_, Vol_poor_ and Vol_not_ represent the amount in mL of normally, poorly and not aerated lung over the total ROI volume. As previously described for LUS evaluation, changes over time of lung CT densities (ΔCT_air_) were calculated as follows:

$$\Delta {\text{CT}}_{{{\text{air}}}} = {\text{ P}}_{{{\text{air}}\,\left( {{\text{Late}}} \right)}} {-}{\text{ P}}_{{{\text{air}}\,\left( {{\text{Early}}} \right)}}$$

where (Late) and (Early) were the same time points as previously described.

Lung aeration improved if ΔCT_air_ was greater than 0%, whereas lung aeration did not change or worsened if ΔCT_air_ was equal to or less than 0%. Changes over time of P_norm_, P_poor_ and P_not_ in each ROI were calculated as follows:

$$\Delta {\text{CT}}_{{{\text{norm}}}} = {\text{ P}}_{{{\text{norm}}\,\left( {{\text{Late}}} \right)}} {-}{\text{ P}}_{{{\text{norm}}\,\left( {{\text{Early}}} \right)}}$$

$$\Delta {\text{CT}}_{{{\text{poor}}}} = {\text{ P}}_{{{\text{poor}}\,\left( {{\text{Late}}} \right)}} {-}{\text{ P}}_{{{\text{poor}}\,\left( {{\text{Early}}} \right)}}$$

$$\Delta {\text{CT}}_{{{\text{not}}}} = {\text{ P}}_{{{\text{not}}\,\left( {{\text{Late}}} \right)}} {-}{\text{ P}}_{{{\text{not}}\,\left( {{\text{Early}}} \right)}}$$

where (Late) and (Early) were the same time points as previously described. Changes over time of P_air_, P_norm_, P_poor_ and P_not_ for the entire lung were calculated as the median of ΔCT_air_, ΔCT_norm_, ΔCT_poor_ and ΔCT_not_ obtained in all the twelve ROI, respectively. Similarly, changes over time of P_air_, P_norm_, P_poor_ and P_not_ for the ventral, intermediate and dorsal zones were calculated as the median of ΔCT_air_, ΔCT_norm_, ΔCT_poor_ and ΔCT_not_ obtained in all the four ventral intermediate and dorsal ROI, respectively.

### Statistics

Data were tested for normal distribution by Shapiro–Wilk test and were expressed as mean and standard deviation (SD) or median with interquartile range 25–75 (IQR), as appropriate.

The relationship between median ΔCT_air_, ΔCT_norm_, ΔCT_poor_, ΔCT_not_ and ΔLUS was evaluated overall and in ventral, intermediate and dorsal regions by Pearson’s correlation coefficient with a null hypothesis that the correlation coefficient was equal to zero; p < 0.05 was considered statistically significant. Comparison between the early and late phase of ARDS was performed with Wilkcoxon test for paired samples. Kruskal–Wallis one-way analysis of variance with post-hoc Dunn’s test for multiple comparison was performed to compare ΔCT_air_, ΔCT_norm_, ΔCT_poor_, ΔCT_not_ between categorical changes of ΔLUS. To obtain an 80% power to detect a negative correlation between ΔLUS_tot_ and ΔCT_air_ (primary endpoint) of at least − 0.75, with an alpha error of 0.05 (one sided), a sample size of 10 participants was required. Statistical analyses were performed using Stata 13.1/SE (Stata Corporation, Texas, USA).

## Results

### Study population

Eleven patients with ARDS were enrolled in the study. Twenty-two lung CT scans were performed corresponding to twenty-two LUS examinations. A total of 132 LUS fields for each time point (Early VS Late) were evaluated, corresponding to an equal number of CT ROIs. Each ventral, intermediate and dorsal region encompassed a total of 44 fields/ROIs. Median age was 52 years (44–58), 7 (64%) were male, Severity Acute Physiologic Score (SAPSII) and Sequential Organ Failure Assessment (SOFA) score at ICU admission were 42 (34–51) and 10 (8–11), respectively. ARDS aetiology was bacterial pneumonia in 6 (55%), viral pneumonia in 4 (36%) and abdominal sepsis in 1 (9%) patients. Five (45%) and six (55%) patients over eleven died after 28 and 60 days from ICU admission, respectively. ICU length of stay was 29 (19–45) days. Clinical data of the included patients at Early and Late timepoints are reported in Table 1S.

### Monitoring of lung aeration over time

**Fig. 1:**
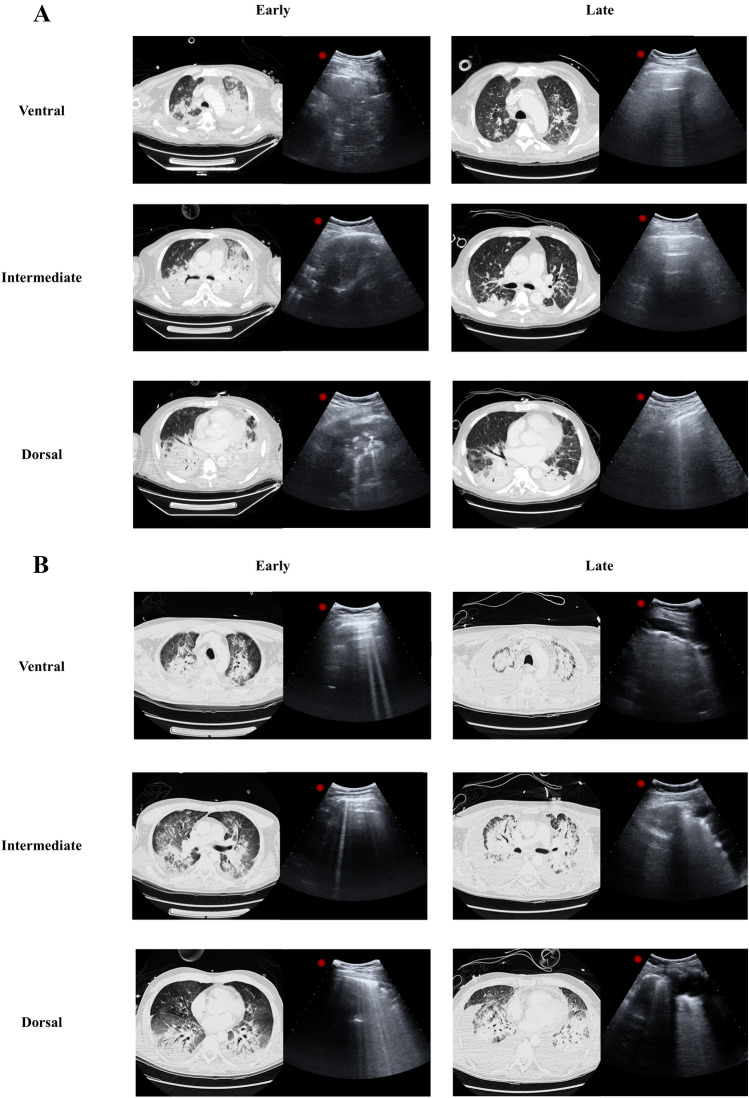
Representative lung CT and corresponding LUS images in ventral, intermediate and dorsal lung regions at early and late stages of ARDS, in two opposite cases of aeration improvement (panel **A**) or worsening (panel **B**)

Representative lung CT and corresponding LUS images in ventral, intermediate and dorsal lung regions at early and late stages of ARDS in two opposite cases of aeration improvement or worsening are shown in Fig. 1 (panel A and B). Absolute total and regional LUS score and P_air_, P_norm_, P_poor_ and P_not_ values at Early and Late time points are described in Table 1S.

Correlations between median ΔCT_air_, ΔCT_norm_, ΔCT_poor_, ΔCT_not_ and ΔLUS (N = 11) are described in Table 1. Increments in ΔLUS were significantly inversely related to median ΔCT_air_ decrease both globally (ΔLUS_tot_ r = − 0.74, p < 0.01) and in ventral (ΔLUS_V_ r = − 0.66, p < 0.05), intermediate (ΔLUS_I_ r = − 0.69, p < 0.05) and dorsal (ΔLUS_D_ r = − 0.63, p < 0.05) regions. Similarly, ΔLUS was positively correlated to median ΔCT_not_ both globally (ΔLUS_tot_ r = 0.79, p < 0.01) and in ventral (ΔLUS_V_ r = 0.76, p < 0.01), intermediate (ΔLUS_I_ r = 0.74, p < 0.01) and dorsal (ΔLUS_D_ r = 0.62, p < 0.05) regions. Median ΔCT_poor_ showed poor or no correlations with corresponding LUS variations. Median ΔCT_norm_ was significantly associated to ΔLUS_tot_ and ΔLUS_I_ (r = − 0.67, p < 0.05 and r = − 0.66, p < 0.05, respectively).

**Table 1:** Pearson’s correlations between median ΔCT_air_, ΔCT_norm_, ΔCT_poor_, ΔCT_not_ and ΔLUS

		ΔCT_air_	ΔCT_norm_	ΔCT_poor_	ΔCT_not_
Overall					
ΔLUS_tot_					
r		− 0.74	− 0.67	− 0.13	0.79
p		< 0.01	< 0.05	0.71	< 0.01
Ventral regions					
ΔLUS_V_					
r		− 0.66	− 0.58	− 0.18	0.76
p		< 0.05	0.06	0.59	< 0.01
Intermediate regions					
ΔLUS_I_					
r		− 0.69	− 0.66	0.20	0.74
p		< 0.05	< 0.05	0.56	< 0.01
Dorsal regions					
ΔLUS_D_					
r		− 0.63	− 0.55	0.01	0.62
p		< 0.05	0.09	0.98	< 0.05

*r* Pearson’s correlation coefficient, Δ*LUS*_*tot*_ the difference between the sum of the LUS scores obtained in every 12 fields at T late and at T early, Δ*LUS*_*V*_ the difference between the sum of the LUS scores obtained in the four ventral fields at T late and at T early, Δ*LUS*_*I*_ the difference between the sum of the LUS scores obtained in the four intermediate fields at T late and at T early, Δ*LUS*_*D*_ the difference between the sum of the LUS scores obtained in the four dorsal fields at T late and at T early

Changes over time of P_air_, P_norm_, P_poor_ and P_not_ for the entire lung were calculated as the median of ΔCT_air_, ΔCT_norm_, ΔCT_poor_ and ΔCT_not_ obtained in all the twelve ROI, respectively

**Fig. 2:**
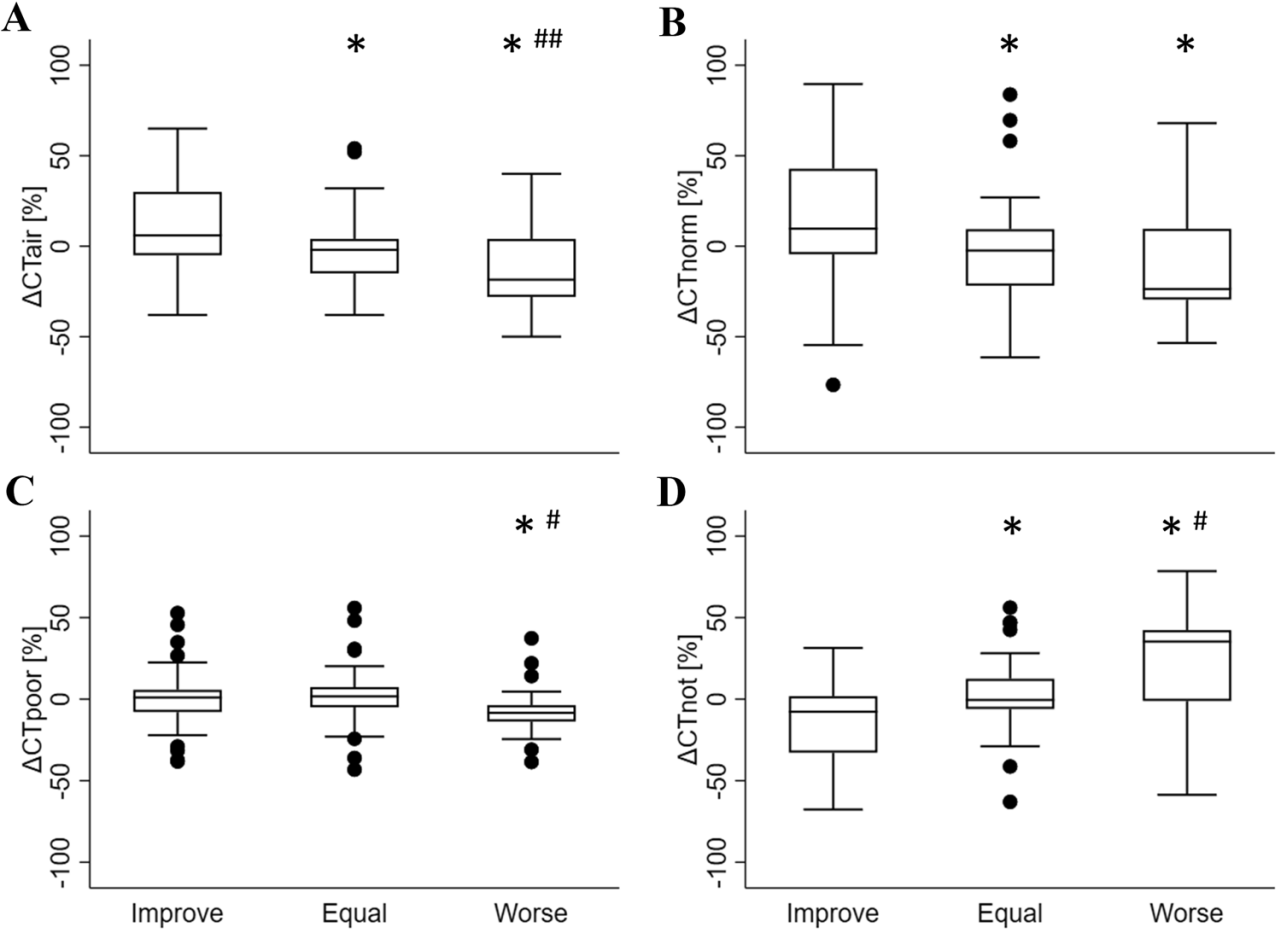
Changes in percentage of aerated tissue (**A**) and changes in normally (**B**), poorly (**C**) and not (**D**) aerated tissue over categorical changes in LUS score. *p < 0.01 VS “Improve” category, #p < 0.01 VS “Equal” category, ##p < 0.05 VS “Equal” category

Lung CT density variations, in Early and Late time points over categorical changes in LUS score are shown in Table 2. In Fig. 2 the changes in median ΔCT_air_, ΔCT_norm_, ΔCT_poor_ and ΔCT_not_ are shown based on the corresponding changes in categorical ΔLUS score for each field. Percentage of aeration at CT scan significantly reduced in the fields where lung aeration worsened according to LUS score [19% (− 28–4)], compared to the fields in which LUS score remained the same [− 2% (− 15–4), p < 0.0385] or improved [6% (− 5–30), p = 0.0000] and in the fields in which LUS score remained the same [− 2% (− 15–4)] compared to the ones in which LUS score improved [6% (− 5–30), p = 0.0029–panel A]. Similarly, there was a significant increase in terms of amount of ΔCT_not_ in the fields where LUS score worsened [35% (− 1–42)] compared to the fields in which LUS score remained the same [− 1% (− 6–13), p = 0.0049] or improved [− 8% (− 32–2), p = 0.0000] and in the fields in which LUS score improved [− 8% (− 32–2)] compared to the ones where LUS score remained the same [− 1% (− 6–13), p = 0.0043–panel D]. Increments in ΔCT_norm_ were significant only in the fields where LUS score improved (10% (− 4–43)) compared to the ones in which LUS score remained the same [− 2% (− 22–9), p = 0.0030] or worsened [− 24% (− 30–19), p = 0.0000–panel B]. Conversely, a reduction in ΔCT_poor_ was statistically significant only in the fields where LUS score improved [1% (− 8–6), p = 0.0020] or remained the same [2% (− 5–7), p = 0.0002] compared to the fields where LUS score worsened [− 8% (− 14–4)–panel C].

**Table 2:** Lung CT density in Early and Late stages of ARDS over categorical changes in LUS score

Variables	Improve		Equal		Worse	
	Early	Late	Early	Late	Early	Late
	N = 49	N = 49	N = 45	N = 45	N = 34	N = 34
P_air_ (%)	34 (15–57)	52 (37–60)*	42 (29–55)	38 (27–54)	38 (31–45)	17 (10–41)*
P_norm_ (%)	43 (6–61)	60 (38–75)*	43 (27–62)	35 (22–59)	40 (27–47)	15 (8–35)*
P_poor_ (%)	23 (15–31)	20 (14–30)	28 (20–38)	34 (18–45)	30 (24–50)	23 (16–40)*
P_not_ (%)	30 (11–51)	12 (8–25)*	20 (10–32)	19 (12–35)	26 (14–32)	55 (20–67)*

“Improve”, “Equal” or “Worse”: ΔLUS < 0, = 0 or > 0, respectively

*Early* study entry, *Late* at least 1 week after T early, *N* number of CT regions of interest involved, *Improve* fields in which LUS score improved from T early to Late, *Equal* fields in which LUS remained the same from T early to Late, *Worse* fields in which LUS score worsened from T early to Late, *Pair* percentage of aeration, *P*_*norm*_*, P*_*poor*_*, P*_*not*_ percentage of over, normally, poorly and not aerated lung, respectively

*p < 0.01

The Spearman correlation coefficient field by field between ΔLUS and ΔCTair was – 0.42 (p < 0.0001, n = 128). Spearman correlation coefficient between ΔLUS and ΔCTnorm and ΔCTnot were – 0.40 (p < 0.05) and 0.48 (p < 0.0001), respectively.

## Discussion

The main findings of this study is that LUS had a good correlation with lung computed tomography analysis in detecting changes of lung aeration when performed at early and late stages of ARDS. Improving or worsening of LUS was associated with higher percentage of normally and not aerated regions of lung computed tomography, respectively. Recently, Chiumello et al. showed that LUS is a valuable bedside tool to evaluate lung tissue aeration, although it should probably not be used to assess alveolar recruitment [5]. In ARDS patients supported with extra-corporeal membrane oxygenation (ECMO), LUS has shown to be a valid tool for daily monitoring of aeration [20]. In addition, lung ultrasound has been demonstrated to be a useful method to evaluate changes in extravascular lung water in patients with ARDS [21] and acute kidney injury requiring renal replacement therapy and pulmonary congestion of patients with chronic heart failure [22]. The number of B lines was also associated with net negative fluid balance after dialysis [22] and with hospital length of stay and mortality in a cohort of ambulatory heart failure patients [23]. Recently, LUS performed directly on the lung surface has been used to monitor lung aeration changes over time and as a prognostic tool in lung donor in the context of the normothermic ex-vivo lung perfusion (EVLP) [24, 25]. Our study highlights for the first time that LUS, compared with the gold standard CT, was able to detect even small changes in percentage of aeration (ΔCT_air_) over time, suggesting that LUS may represent an accurate bedside and radiation-free tool to monitor and quantify the degree of ARDS resolution or worsening. In addition, our study shows that LUS performed better in recognizing changes of not aerated (ΔCT_not_) tissue. This finding could be explained with the fact that LUS accuracy relies basically on the presence/absence of B lines or consolidation, which are predominant in not aerated tissue. Resolution of consolidation pattern (e.g. from consolidation to few B lines or A lines) is easily recognized by LUS. Regarding LUS performance in recognizing changes in normally aerated tissue, a possible explanation consists in the fact that A lines artifact is the hallmark for normally aerated tissue, and the emergence of B lines is easily identified without much false positive. Conversely, it is impossible for LUS to distinguish between overinflated tissue and normal aeration. At the same time, comparing a categoric ordinal variable as LUS score with a continuous one, is probably the rationale to explain the limitation of LUS in discriminating the faceted aspects of a poorly aerated slice.

Following up ARDS radiological changes could be challenging in the ICU because of the low accuracy of bedside anterior–posterior chest X-ray in defining ARDS morphology and the distribution of lung aeration loss [26, 27]. CT scan remains the gold standard for monitoring lung aeration changes, but it could be harmful in daily practice because of the risks related to patient mobilization and ionized radiations. Patients with severe ARDS can be burdened during the transfer from the ICU to the CT scan facility by several adverse events which might compromise oxygenation, such as accidental disconnection from the ventilator with lung de-recruitment. In addition, patients with ECMO support can be exposed to severe complications such as cannulas misplacement and pump malfunction that negatively contribute to patient’s outcome. Lung ultrasound might represent therefore an accurate and cost effective bedside tool for monitoring lung aeration changes over time. In addition, the quantitative lung aeration assessment with dedicated software that we used in this study (e.g. Maluna® or analogues) could not be widely available in every ICU, thus limiting CT scan efficacy in evaluating lung parenchyma aeration in daily practice.

This study has some limitations. First, the different spatial resolution of CT and LUS may influence lung aeration assessment [6]. In fact, different from LUS, CT scan analysis encompasses the whole thoracic area from along the chest wall to mediastinal organs. Second, this study was a pilot study with a small sample size; further external validation is warranted. Third, the whole analyses were performed on ARDS patients before the COVID-19 pandemic. Therefore, we can’t extend our findings to patients with COVID-19 associated ARDS. Fourth, B-lines were quantified by real time counting, without using have automatic B line quantification. This might have affected overall accuracy [28].

## Conclusions

In conclusion, in our cohort of ARDS patients, LUS was a reliable bedside tool to monitor changes of lung aeration overtime when compared to the gold standard lung computed tomography. This technique might be employed in patients with ARDS to reliably assess the healing or not of the lung parenchyma. Future research is required to confirm the utility of this technique in larger studies.

## Supplementary Information

Below is the link to the electronic supplementary material.

Supplementary file1 (DOCX 16 KB)
