# Correction: Clinical performance of lung ultrasound in predicting time-dependent changes in lung aeration in ARDS patients

**DOI:** 10.1007/s10877-024-01137-2

**Published:** 2024-03-16

**Authors:** Andrea Costamagna, Irene Steinberg, Emanuele Pivetta, Pietro Arina, Simona Veglia, Luca Brazzi, Vito Fanelli

**Affiliations:** 1 Department of Anaesthesia and Critical Care, AOU Città Della Salute E Della Scienza Di Torino, University of Turin, Corso Dogliotti 14, 10126 Turin, Italy; 2 Department of Surgical Sciences, University of Turin, Turin, Italy; 3 Division of Emergency Medicine and High Dependency Unit, Department of Medical Sciences, AOU Città Della Salute E Della Scienza Di Torino, Turin, Italy; 4 Division of Medicine, UCL, Bloomsbury Institute for Intensive Care Medicine, Gower Street, London, UK; 5 Department of Diagnostic Imaging and Radiotherapy, AOU Città Della Salute E Della Scienza Di Torino, University of Turin, Turin, Italy

**Correction to: Journal of Clinical Monitoring and Computing** 10.1007/s10877-022-00902-5

In the original version of this article, the given and family names of all the authors were swapped incorrectly as Costamagna Andrea · Steinberg Irene · Pivetta Emanuele · Arina Pietro · Veglia Simona · Brazzi Luca · Fanelli Vito. The corrected given and family names of the authors are given in this Correction. The original article has been updated.

