# Supplementary material for: Clinical performance of lung ultrasound in predicting time-dependent changes in lung aeration in ARDS patients
Source: J Clin Monit Comput. 2022 Aug 8;37(2):473–80. doi: 10.1007/s10877-022-00902-5 (PMC9358118; doi:10.1007/s10877-022-00902-5)
Supplement: Supplementary file 1 — Supplementary file1 (DOCX 16 KB) [file 10877_2022_902_MOESM1_ESM.docx]

**Table 1S.** Clinical data and LUS score in Early and Late timepoints.

| **Variables** | **Early (N=11)** | **Late (N=11)** |
| --- | --- | --- |
| ARDS severity N(%)  Mild  Moderate  Severe | 2 (18)  1 (9)  8 (73) | 3 (27)  4 (36)  4 (36) |
| SOFA | 10 (8 – 11) | 6 (5 – 8)* |
| Ventilatory mode N(%)  PSV  IPPV (VCV or PCV) | 1 (9)  10 (91) | 4 (36)  7 (64) |
| Vt/PBW | 5.8 (4.8 – 6.9) | 6.9 (5.7 – 7.2) |
| PEEP tot (cmH_2_O) | 15 (13 – 16) | 8 (6 – 12)* |
| PaO_2_/FiO_2_ (mmHg) | 153 (98 – 228) | 171 (143 – 290) |
| vv-ECMO N(%) | 6 (55) | 3 (27) |
| LUS_TOT_ | 23±7 | 20±9 |
| LUS_V_ | 6±2 | 5±3 |
| LUS_I_ | 8±3 | 7±4 |
| LUS_D_ | 10±2 | 9±3 |
| Pair | 39±18 | 41±17 |
| Pnorm | 37±21 | 43±26 |
| Ppoor | 29±15 | 27±15 |
| Pnot | 28±17 | 26±22 |
|  |  |  |

**List of abbreviations.** Early: study entry; Late: at least one week after T early; N: number of patients. ARDS: acute respiratory distress syndrome; SOFA: Sequential Organ Failure Assessment score; vv-ECMO: venous-venous extra corporeal membrane oxygenation; PSV: pressure-support ventilation; IPPV: intermittent positive pressure ventilation; VCV: volume-controlled ventilation; PCV pressure-controlled ventilation; Vt: tidal volume; PBW: predicted body weight; PEEP: positive end-expiratory pressure; PaO2/FiO2: ratio of the arterial oxygen partial pressure to fraction of the inspired oxygen; LUS_TOT_: total lung ultrasound score; LUS_V_: ventral regions LUS; LUS_I_: intermediate regions LUS; LUS_D_ dorsal regions LUS; Pair: percentage of aeration; Pnorm: percentage of normally aerated lung; Ppoor percentage of poorly aerated lung; Pnot: percentage of not aerated lung. *=p<0.01 vs Early
